# Double-Stranded RNA-Based Method for Diagnosing Severe Fever with Thrombocytopenia

**DOI:** 10.3390/jcm14010105

**Published:** 2024-12-28

**Authors:** Jung Wan Park, Jaemin Jeon, Yoosik Kim, Min Hyok Jeon

**Affiliations:** 1 Department of Internal Medicine, Division of Infectious Disease, Soonchunhyang University Hospital, Cheonan 31151, Republic of Korea; splendidmagic@schmc.ac.kr; 2 Department of Chemical and Biomolecular Engineering, Korea Advanced Institute of Science and Technology, Daejeon 34141, Republic of Korea

**Keywords:** double-stranded RNA, SFTS, tick-borne diseases, spiropyran-based method, RNA extraction methods, molecular diagnostics

## Abstract

**Background/Objectives**: This study explores the potential of using elevated levels of blood double-stranded RNA (dsRNA) as a diagnostic tool for severe fever with thrombocytopenia syndrome (SFTS) infection. **Methods**: Blood samples from SFTS patients were collected, dsRNA was purified, and total dsRNA expression was quantitatively analyzed using a spiropyran-based method. Comparative analysis was performed using blood samples from healthy individuals and scrub typhus patients with similar symptoms. **Results**: The results revealed that individuals infected with SFTS had significantly higher total blood dsRNA levels compared to healthy or scrub typhus controls. The dsRNA-based method also has potential for assessing infection severity based on dsRNA levels. **Conclusions**: These findings suggest that total dsRNA expression can serve as a quick and convenient method to differentiate SFTS from other non-viral conditions with similar clinical presentations. This method shows promise as a novel diagnostic tool.

## 1. Introduction

Severe Fever with Thrombocytopenia Syndrome (SFTS) is a viral infectious disease caused by the bite of ticks, such as *Haemaphysalis longicornis* (small cattle blood tick), which carry the SFTS virus. It poses a significant health risk with a high mortality rate of 20%. Clinically, after an incubation period of 1–2 weeks, patients typically present within 7 days of onset with high fever, thrombocytopenia, and in about one-third of cases, sepsis with leukopenia, decreased consciousness, and multiple organ failure. This clinical deterioration is associated with a rapid increase in blood cytokines, including interleukin (IL)-10, IL-6, interferon (IFN)-α, IFN-γ, and interferon gamma-induced protein-10 [1,2].

SFTS was first reported in China in 2011 [3] and has since been increasingly reported in East Asia, including Korea and Japan [4]. In South Korea, the incidence has risen annually since the first case was identified in 2013, with 243 cases reported in 2020. The number of SFTS patients continues to increase, and the distribution of *H. longicornis*, the vector of SFTS, is gradually expanding [5,6], suggesting that SFTS will spread further. However, no proven treatment with clinical benefit beyond conservative care poses a significant challenge for physicians treating SFTS patients [7,8].

The clinical symptoms and blood test abnormalities of SFTS patients are similar to those of other fall febrile illnesses, such as scrub typhus and hemorrhagic fever with renal syndrome (HFRS), making the diagnosis clinically challenging. Therefore, genetic detection of the causative virus is essential to confirm the diagnosis. However, under current management guidelines, SFTS can only be diagnosed by polymerase chain reaction (PCR) testing performed by local health and environmental laboratories. Frontline healthcare centres are not equipped with PCR testing methods, resulting in an average of 3 to 5 days for a patient’s blood sample to be transported to a local health and environmental laboratory, tested, and finally diagnosed with SFTS [9]. There is concern among experts that delayed diagnosis can lead to late treatment, such as plasma exchange, which can adversely affect patient outcomes [10]. Particularly, since 2012, infections have been reported in healthcare workers who have been exposed to the blood of SFTS patients, and there is a risk of increased healthcare worker infections due to lack of proper protective gear when clinical diagnosis of SFTS infection is delayed [11]. Therefore, there is a need for a diagnostic technology that can be used to diagnose SFTS more quickly.

Double-stranded RNA (dsRNA) is a key activator of the innate immune response during viral infections. dsRNA is associated with most viral infections, either as the viral genome (in the case of dsRNA viruses) or as a product of viral replication (for single-stranded RNA (ssRNA) and DNA viruses) in infected cells [12]. Most host-derived RNA is single-stranded, while nearly all RNA viruses, including hepatitis C virus, influenza virus, and SARS-CoV-2, form dsRNA during replication. Therefore, experts suggest that detecting dsRNA could be an effective method for fast and universal identification of possible viral infections [13]. A method for detecting viruses using dsRNA sequencing in hepatitis viruses has been proposed [14]. Additionally, another study showed that detecting dsRNA using a kit as a diagnostic method for viral infections was not only faster than conventional PCR, but also had the advantage of not requiring specialized testing equipment or testing experts [15]. However, there are still no reports on the use of dsRNA as a diagnostic method for viral infections, and studies specifically related to the diagnosis of SFTS are lacking. Therefore, we aimed to determine whether dsRNA can be detected in the serum of SFTS patients and if it can be used for diagnosis.

## 2. Materials and Methods

### 2.1. Selection of Patient Cohorts

Among the patients who visited Soonchunhyang University Hospital in Cheonan, South Korea, from January 2022 to December 2023, those who were confirmed to have SFTS by PCR and gave their consent were selected as subjects. For comparative analysis, blood samples from random patients who visited the health checkup centre of the same medical institution during the same period and had residual blood samples from their checkups were selected as normal controls. To confirm that the dsRNA test can differentiate from other diseases, patients diagnosed with scrub typhus who visited the same medical institution during the same period were selected as abnormal controls after receiving consent. Blood samples and clinical information were collected from these patients.

The study protocol received approval from the Institutional Review Board of Soonchunhyang University Cheonan Hospital (IRB No. 2021-11-041., registered on 17 November 2021), and followed the ethical guidelines of the World Medical Association Declaration of Helsinki. Informed consent was obtained from all individual participants included in the study. This study adhered to the Strengthening the Reporting of Observational Studies in Epidemiology (STROBE) checklist.

### 2.2. Blood Specimen Collection and Pretreatment

A total of 10 mL of blood was collected in EDTA tubes after obtaining informed consent from both the patients and abnormal controls. The collected samples were centrifuged to separate the serum and blood cell layers. We centrifuged using BD vacuum tube for serum and heparin tube for plasma. After centrifugation at 2000× *g* for 15 min at 4 °C, only the supernatant was collected in a 1.5 mL tube. Serum was pretreated with RNA later™ Stabilisation Solution^®^ (Thermo Fisher Scientific Inc., Waltham, MA, USA) at a 1:1 ratio, and blood cell layers were pretreated with TRIzol™ Reagent^®^ (Invitrogen, Waltham, MA, USA). All samples were then snap frozen in liquid nitrogen (−197 °C) and stored in an ultra-low temperature freezer below −70 °C. For asymptomatic control patients, due to the limited residual sample volume, the serum obtained after centrifugation was pretreated with RNA later^®^ and stored in a −70 °C deep freezer.

### 2.3. RNA Extraction from Trizol-Treated Samples

Total RNA was extracted using TRIzol^®^ (Invitrogen, Waltham, MA, USA) following the phenol/chloroform method. DNA contamination was removed using DNase I^®^ (Takara, Japan), and the purified RNA was then processed using the oligo Clean & Concentrator^®^ (Zymo Research, Irvine, MA, USA) [16].

### 2.4. Extraction of RNA

Scrub typhus sample was not a trizol treated sample but an RNA later^®^ treated whole blood sample. RNA was isolated using the RiboPure™ RNA Purification Kit^®^ (Thermo Fisher Scientific Inc., USA). One milliliter of each blood sample was preserved in RNA later^®^ and stored at below −70 °C as per the manufacturer’s instructions. Upon thawing, RNA later^®^ was separated by centrifugation, followed by RNA extraction and DNase treatment according to the manufacturer’s protocol.

In all experiments we performed, RNA was concentrated to 20 ng/μL, and 10 μL was used for measurements.

### 2.5. Spiropyran Assay

**Figure 1:**
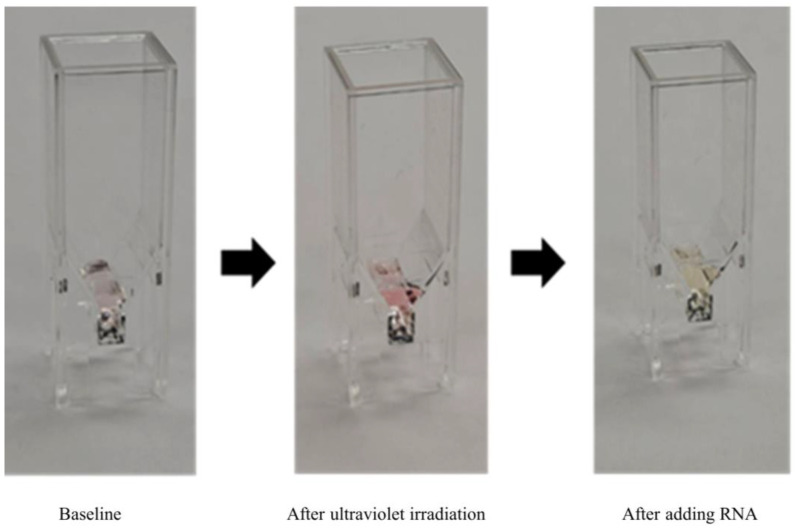
Spiropyran test. The assay utilizes spiropyran (SP), which exists in two forms—spiropyran (SP) and merocyanine (MC)—when dissolved in water (with SP being the predominant form). The SP is colourless, while MC has a reddish tint due to its altered ring structure. Upon UV irradiation, SP isomerizes to MC, resulting in a deep red colour. MC can intercalate between RNA base pairs and further convert to protonated form (MCH+), which appears yellowish. The absorbance measurements before and after sample treatment allow quantification of dsRNA.

Amidine-conjugated spiropyran (Am-SP) was dissolved in triple distilled water TDW to achieve a concentration of 12 mM. Am-SP was converted to its open amidine merocyanine (Am-MC) moiety with an absorbance of 0.080 at 515 nm, by exposing it to 254 nm ultraviolet light for 1 min using a UV lamp (7 mW/cm^2^). All experiments were conducted in darkness to prevent reverse isomerization of Am-MC to Am-SP (Figure 1) [17,18]. Ten microliter sample of RNA was pipetted directly into a cuvette containing Am-MC and incubated. Moreover, absorbance was measured within 1 min using a spectrophotometre (Eppendorf, USA) with a 1 mm path length. Absorbance readings were baseline-corrected and background-corrected to establish consistent isosbestic points at 395 nm and 480 nm. The rate of absorbance change at 515 nm was calculated using the formula: (sample-control)/control × 100 (%). The control solution was TDW, a solvent for RNA.

The wavelength range measured by a spectrophotometer is 350–700 nm. To find the isosbestic point, you can plot several absorption spectra of the sample at different stages of the reaction or physical change and find the point where all the spectra intersect [18].

### 2.6. SFTS Quantitative Real-Time PCR (qPCR)

The qPCR for SFTS was performed by a national organization (Chungcheongnam-do Institute of Health and Environment, Hongseong-gun, Republic of Korea) following the national policy. qPCR was performed at the institute using Thermofisher Quant studio 5 equipment (Thermo Fisher Scientific Inc., USA) and Kogenebiotech IG5010S reagents (Kogenebiotech, Seoul, Republic of Korea). This kit targets the M and S segments of the SFTS virus, and we determined the viral RNA copy number by standard curve amplification of positive control RNA and set the cutoff cycle threshold (Ct) for positivity to 35 cycles. If the Ct value of a sample is lower than the cutoff, it is considered positive.

### 2.7. Statistical Analysis

Statistical analysis was performed using SPSS version 21.0 for Windows (IBM Corporation, Armonk, NY, USA, released 2012). Continuous variables were analysed using the Mann-Whitney U test. A *p*-value of less than 0.05 was considered statistically significant for two-tailed tests.

## 3. Results

We initiated the investigation with five blood samples from SFTS patients, 50 normal control blood samples, and 10 abnormal control blood samples. Samples were excluded if clotting occurred during processing and RNA extraction could not be completed. Absorbance analysis indicated that RNA was incorrectly extracted in cases where absorbance decreased less than when water was added, leading to their exclusion from the dataset. Consequently, a total of three SFTS samples, 12 normal control samples, and 2 abnormal control samples were ultimately used in the experiment.

### 3.1. Demographics, Clinical Symptoms, Serologic Tests, and Clinical Outcomes of SFTS Patients

The clinical findings of the three patients who tested positive by QPCR were as follows: The mean age was 74 years. Two patients had a history of regular field work, while one did not have a specific history of outdoor activities, making their exposure to small cattle ticks unclear. Upon admission, all three patients exhibited increased body temperature but stable other vital signs. However, during inpatient treatment, symptoms such as decreased consciousness and decreased oxygen saturation worsened, necessitating intensive care unit management. Blood tests at admission revealed leukopenia, particularly lymphopenia, thrombocytopenia, elevated liver enzymes, increased lactic dehydrogenase (LDH), elevated ferritin, elevated adenosine deaminase (ADA), and prolonged activated partial thromboplastin time. Two patients recovered without significant complications, but one patient succumbed 4 days after hospitalisation (Table 1).

### 3.2. SFTS qPCR

The cycle threshold values (Ct values) for SFTS patients were determined to be 20.37, 23.78, and 21.41 for the M segment, and 23.14, 22.32, and 23.20 for the S segment, respectively.

### 3.3. SFTS vs. Normal Controls vs. Abnormal Controls (Scrub Typhus)

The dsRNA expression was analysed using an in-house developed method using Am-MC, which detects changes in absorbance spectra due to intercalation between complementary RNA pairs. Previous studies have demonstrated the method’s ability to quantitatively assess total dsRNA expression following drug treatments [16,18]. Importantly, the spiropyran-based method exhibited superior performance compared to conventional approaches using the J2 antibody, which specifically targets dsRNA longer than 40 bp.

**Figure 2:**
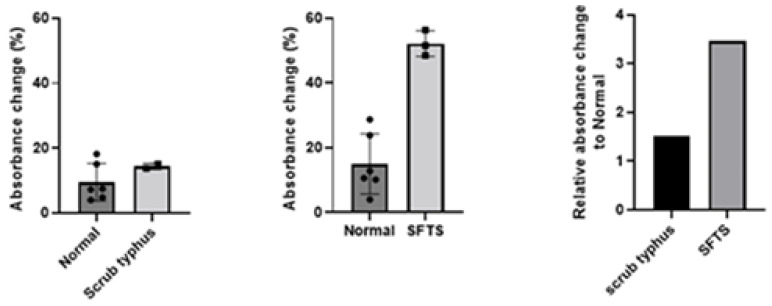
Absorbance difference between SFTS, normal control, and abnormal control (Scrub typhus).

The absorbance measurements for dsRNA in SFTS patients averaged 52.12 ± 3.94% (individual values: 56.31%, 48.49%, and 51.54%, respectively). In contrast, the absorbance in the normal control group averaged 12.23 ± 7.61%, and in the abnormal control group, it averaged 14.42 ± 0.93%. There was no statistically significant difference in absorbance between the normal and abnormal control groups (*p* = 0.296). However, the absorbance differed significantly between the normal control group and the SFTS group (*p* < 0.001). In summary, the relative absorbance changes of SFTS and abnormal control sera compared to normal control sera were nearly 2-fold higher (1.52 vs. 3.47) (Figure 2).

## 4. Discussion

To broaden the choice of diagnostic methods for SFTS, our study aimed to evaluate the potential of using total dsRNA level to identify SFTS infection. We sought to determine the elevation of dsRNA in the blood of SFTS patients compared to normal controls and assess its diagnostic value in differentiating SFTS from scrub typhus, a representative vector-borne zoonotic disease in South Korea. The abnormal control was set to a patient with Tsutsugamushi for the following reasons: (1) The disease epidemic season is in autumn, when Tsutsugamushi and SFTS are similar, and clinically, the symptoms are similar, so it is not easy to differentiate between the two diseases at the time of presentation to the hospital; (2) However, the prognosis of the two diseases is different. Tsutsugamushi can be treated with doxycycline and has a low mortality rate, while SFTS has no cure and a high mortality rate.

dsRNA, a byproduct of viral replication, usually consists of several hundred to thousands of bp and is known to be a major activator of the innate immune response to viral infections [19]. Furthermore, the production of dsRNA during viral genome replication is thought to be shared by most viruses. Immunofluorescence assay (IFA) identification of dsRNA expressed by viruses such as adenovirus, encephalomyocarditis virus, influenza A virus, herpes simplex virus, La Crosse virus, SARS-CoV-2, and others is detectable in positive ssRNA viruses and DNA viruses, but lower in negative ssRNA viruses [20]. Upon viral infection, dsRNA is recognised by a pattern recognition receptor that stimulates the expression of type I IFNs (e.g., IFNα/β). The expressed cytokines are secreted into the extracellular space and stimulate IFN receptors, activating the JAK/STAT signaling pathway to upregulate the expression of IFN-stimulated genes [21]. This, in turn, inhibits the synthesis of antiviral effector proteins. A classic example is COVID-19 infection. SARS-CoV-2-derived dsRNAs mediate the innate immune response in respiratory epithelial cells and cardiomyocytes, which subsequently triggers a hyperinflammatory syndrome, such as a cytokine storm, and can lead to serious complications in the lungs and heart. These pieces of evidence suggest that the evasion of the immune response by dsRNAs ultimately contributes to the unique pathogenesis of COVID-19 [22].

Detection of dsRNA for the diagnosis of viral infections has been attempted in the past. In a study of respiratory viruses, detection of anti-dsRNA in respiratory specimens using immunofluorescence was found to have a sensitivity of 83.3%, specificity of 87.4%, positive predictive rate of 89.1%, and negative predictive rate of 80.9% [23]. We have also shown that detecting viral dsRNA may serve as a universal diagnostic platform to quickly assess infectious status [15]. Although not studied in humans, the identification of dsRNA in Usutu virus outbreaks in birds has been used to identify the virus lineage. Particularly, identifying dsRNA in individuals with clinically suspected viral infections, where no antigen has yet been identified through testing, can lead to early diagnosis of infection [24]. These studies provide evidence that dsRNA can be utilized as a tool for viral diagnosis.

The link between SFTS and dsRNA has also been demonstrated in several studies. SFTS virus, a member of *Bunyaviridae* family, is a negative ssRNA virus [25]. The pathophysiology of SFTS has been suggested to involve a dsRNA-induced increase in the innate immune response and the generation of a cytokine storm [26]. Studies on the immune response to SFTS have shown that when SFTS infection occurs, viremia is elevated in the body, and dendritic cells are activated. These dendritic cells express Toll-like receptor 3 (TLR 3), which recognises extracellular dsRNAs. When activated, TLR3 rapidly produces large amounts of type I IFNs, leading to the fatal outcome of SFTS infection [27]. Therefore, a large amount of dsRNA is generated during SFTS infection and plays an important role in the pathophysiology of SFTS. Based on this, we hypothesized that detecting dsRNA could be used to diagnose SFTS.

The clinical features of the patients in this study were similar to the general clinical findings of SFTS patients reported in the literature [28]. Following the literature, a cycle threshold Ct value of 39 or less in qPCR was used to diagnose SFTS [29]. Also, it has been reported that viral load differences occur depending on the Ct value, with viral loads exceeding 6 log_10_ copies when the Ct value is between 21 and 25 [30]. In other words, the blood samples were collected when the SFTS virus in each patient was sufficiently multiplying and activating the inflammatory response. Our results clearly showed that SFTS patients exhibited higher total blood dsRNA levels than healthy controls. The elevation of dsRNA, which is not observed in normal controls, indicates the presence of a viral infection and a viral-induced inflammatory response. However, since the normal control group was a randomized sample, the underlying health conditions of these patients were unknown. Some patients may have had conditions that can cause elevated dsRNA levels (e.g., hepatitis B [31], Sjogren’s disease [32]). Further studies are needed to determine if these conditions can be differentiated from SFTS.

Additionally, dsRNA levels were found to be twice as high in samples from SFTS patients compared to normal controls. Co-infection with other pathogens has been reported in approximately 5% of SFTS cases [33], and severe cases of SFTS can pose diagnostic challenges. Consequently, several studies have aimed to distinguish SFTS from other similar diseases [34,35,36]. By employing the proposed dsRNA-based method, diagnosing SFTS could become easier in cases where differentiation from similar diseases is difficult. Early diagnosis of SFTS through dsRNA detection could also facilitate more aggressive intervention, potentially improving patient outcomes.

Similarly, in a study on dsRNA detection in hemorrhagic fever with renal syndrome (HFRS), caused by *Puumala hantavirus*, which shares clinical similarities with SFTS, it was observed that the virus evaded activation of the innate immune response, resulting in minimal dsRNA production [37].

qPCR is a well-established diagnostic method for viral infections that uses gene sequences to determine the presence or absence of a virus. It is highly accurate, and recent studies have shown that it is also faster. However, qPCR requires expensive, specialised equipment and reagents. It is also difficult to tell whether a positive PCR result indicates that the virus is replicating or not. On the other hand, not much is known about dsRNA, but based on our current findings, we believe that it is possible to develop a kit. If further research is carried out, it may be possible to diagnose without the need for specialised equipment or reagents like qPCR. Also, since dsRNA is produced when the virus replicates, the detection of dsRNA may indicate that the virus is multiplying in the body and that the disease is worsening. Therefore, we believe that it is a potentially more informative diagnostic tool than qPCR.

Given dsRNA’s role in viral replication and the development of virus-induced hyperinflammation, future research should explore methods to inhibit dsRNA production as a potential strategy to reduce the high fatality rate of SFTS. Indeed, a previous study has demonstrated the feasibility of separating the two strands of viral dsRNA and inhibiting their transcription or inducing their degradation [38]. Importantly, since human cells only express a limited amount of endogenous dsRNAs, targeting viral dsRNAs holds promise as an agent to inhibit viral replication without affecting normal cellular functions.

This study has several limitations worth noting. First, a limited number of cases were analysed due to restrictions on outdoor activities during the COVID-19 pandemic, resulting in a fewer number of SFTS patients compared to previous years. Additionally, the experimental methods for RNA isolation were not fully established during the study period, leading to fewer tests performed on samples under optimal conditions. Specifically, out of 10 samples collected from patients with scrub typhus, some clotted during pretreatment, which prevented RNA extraction. Second, dsRNA exhibits similarities across most viruses, complicating the identification of specific dsRNA characteristics unique to SFTS and thus calls for the establishment of a dedicated detection method. Nevertheless, we anticipate that these methodological challenges can be addressed through continued research efforts.

## 5. Conclusions

In conclusion, our findings suggest that dsRNA detection holds promise as a fast and convenient diagnostic tool for SFTS. Further studies incorporating larger sample sizes and refining the accuracy and specificity of dsRNA detection for SFTS are warranted and will significantly benefit clinical diagnostics for this fatal disease.

## Figures and Tables

**Table 1:** Clinical baseline characteristics of SFTS patients.

		Patient A	Patient B	Patient C
Sex		Male	Male	female
Age		79	71	73
Farming		+	No	+
Co-existing condition				
	Chronic lung disease	-	-	-
	Chronic heart disease	+	+	-
	Chronic renal disease	-	-	-
	Diabetes mellitus	-	-	-
	Chronic liver disease	-	+	-
	Corticosteroid use	-	-	-
	Cancer	-	-	+
	Cerebrovascular disease	-	-	-
Clinical presentation				
	Fever (Body temperature ≥38.3 °C)	-	+	+
	Headache	-	-	-
	Myalgia	+	-	+
	Anorexia	+	-	+
	Nausea/vomiting	+	-	+
	Abdominal pain	+	-	+
	Diarrhea	-	-	+
	Cough	-	-	-
	Dyspnea	-	-	-
	Decreased consciousness	+	+	+
	Rash	-	-	-
Initial vital sign				
	Blood pressure (mmHg)	150/90	100/60	130/80
	Pulse rate (/min)	130	66	88
	Respiratory rate (/min)	18	15	22
	Body temperature (℃)	37.8	38.4	38
Laboratory findings at admissions				
	WBC count (/μL)	2460	820	980
	Lymphocyte (/μL)	670	140	323
	Hb/Hct (g/dL)	16.5/47.7	17.4/48.3	11.9/34.2
	Platelet count (/μL)	36,000	21,000	33,000
	CPK (U/L)	406	661	864
	LDH (U/L)	1174	1242	1384
	AST (IU/L)	464	490	366
	ALT (IU/L)	133	20	96
	Ferritin (ng/mL)	35,161	37,035	16,355
	PT (INR)	0.96	1.06	0.99
	aPTT (sec)	57.3	54.2	52
	d-dimer (μg/mL)	16.15	17.27	10.75
	CRP (mg/L)	38.26	10.01	3.69
	ADA (IU/L)	189	143	147.6
	Procalcitonin (ng/mL)	0.73	0.544	0.149
	BUN (mg/dL)	21.7	44.2	11.7
	Creatinine (mg/dL)	1.07	1.59	0.79
	Albumin (g/dL)	3.6	4.3	3.6
Outcome				
	Intensive care unit admission	+	+	+
	Time from symptom onset to admission (days)	5	5	5
	Hospital stay day	16	4	22
	Death	-	+	-
SFTS qPCR Ct value				
	M segment	20.374	23.78	21.414
	S segment	23.138	22.317	23.204
dsRNA Absorbance change		56.30954	48.4931	51.54399

## Data Availability

The datasets generated during and/or analysed during the current study are available from the corresponding author on reasonable request.

## Supplementary Materials

The following supporting information can be downloaded at: https://www.mdpi.com/article/10.3390/jcm14010105/s1, Table S1: Baseline characteristics of normal control group; Table S2: Baseline characteristics of abnormal controls (Scrub typhus patients).
