# Supplementary material for: Double-Stranded RNA-Based Method for Diagnosing Severe Fever with Thrombocytopenia
Source: J Clin Med. 2024 Dec 28;14(1):105. doi: 10.3390/jcm14010105 (PMC11721811; doi:10.3390/jcm14010105)
Supplement: Supplementary file 1 [file jcm-14-00105-s001.zip › jcm-3378091-supplementary.pdf]

Supplementary Table S1. Baseline characteristics of normal control group

	Normal Control group
**Sex (male)**	87.5%
**Age**	44.14
**Coexisting condition**	
chronic lung disease	0
chronic heart disease	12.5%
chronic renal disease	0
diabetes	0
chronic liver disease	0
corticosteroid use	0
cancer	0
cerebrovascular disease	25%

Supplementary Table S2. Baseline characteristics of abnormal controls (Scrub typhus patients)

	Scrub typhus patient 1	Scrub typhus patient 2
**Sex**	Male	Female
**Age**	69	59
**Farming**	yes	No
**Coexisting condition**		
chronic lung disease	no	no
chronic heart disease	yes	no
chronic renal disease	no	no
diabetes	no	no
chronic liver disease	no	no
corticosteroid use	no	no
cancer	no	no
cerebrovascular disease	no	no
**Clinical presentation**		
Fever (temperature ≥38.3°C)	yes	yes
Headache	no	yes
myalgia	yes	yes
anorexia	yes	no
nausea/vomiting	no	no
abdominal pain	no	no
diarrhea	yes	no
cough	no	no
dyspnea	no	no
decreased consciousness	no	no

rash	no	no
**Initial vital sign**		
blood pressure	160/100	130/70
pulse rate	70	107
respiratory rate	19	14
body temporature	38.6	37.8
**Laboratory findings at admissions**		
WBC count	6580	5410
lymphocyte	2050	2740
Hb/Hct	14.2/44.0	11.5/34.8
Platelet count	145,000	118,000
LDH	362	519
AST	59	267
ALT	46	159
CRP	11.68	84.71
ADA	130.3	167.9
procalcitonin	0.968	0.240
BUN	11.5	9.2
Creatinine	0.94	0.65
albumin	4.1	4.2
**outcome**		
intensive care unit admission	No	No
time from symptom onset to admission	7	4
hospital stay day	4	6
death	No	No
